# Loneliness as a gender-specific predictor of physical and mental health-related quality of life in older adults

**DOI:** 10.1007/s11136-021-03055-1

**Published:** 2021-12-02

**Authors:** Friederike H. Boehlen, Imad Maatouk, Hans-Christoph Friederich, Ben Schoettker, Hermann Brenner, Beate Wild

**Affiliations:** 1 Department of General Internal Medicine and Psychosomatics, Medical University Hospital Heidelberg, Im Neuenheimer Feld 410, 69120 Heidelberg, Germany; 2 Division of Clinical Epidemiology and Aging Research, German Cancer Research Center, Heidelberg, Germany; 3 Network Aging Research, Heidelberg University, Heidelberg, Germany

**Keywords:** Loneliness, HRQOL, Gender, Older adults

## Abstract

**Purpose:**

Health-related quality of life (HRQOL) in older persons is influenced by physical and mental health, as well as by their social contacts and social support. Older women and men have disparate types of social networks; they each value social ties differently and experience loneliness in unique and personal ways. The aim of this study is, therefore, to determine the longitudinal association between loneliness and social isolation with HRQOL in older people—separated by gender.

**Methods:**

Data stem from the third and fourth follow-up of the ESTHER study—a population-based cohort study of the older population in Germany. A sample of 2171 older women and men (mean age: 69.3 years, range 57–84 years) were included in this study; HRQOL was assessed by using the Short Form-12 questionnaire (SF-12). Data on physical and mental health, loneliness, and social networks were examined in the course of comprehensive home visits by trained study doctors. Gender-specific linear regression analyses were performed to predict physical quality of life (measured by the PCS, physical component score of the SF-12) and mental quality of life (measured by the MCS, mental component score) after three years, adjusted by socioeconomic variables as well as physical, mental, and social well-being.

**Results:**

At baseline, PCS was 41.3 (SD: 10.0) in women and 42.2 (SD: 9.6) in men (*p* = .04). MCS was 47.0 (SD: 10.2) in women and 49.6 (SD: 8.6) in men (*p* < .001). In both genders, PCS and MCS were lower three years later. Loneliness at t0 was negatively associated with both PCS and MCS after three years (t1) among women, and with MCS but not PCS after three years among men. In both genders, the strongest predictor of PCS after three years was PCS at t0 (*p* < .001), while the strongest predictors of MCS after three years were MCS and PCS at t0.

**Conclusion:**

HRQOL in elderly women and men is predicted by different biopsychosocial factors. Loneliness predicts decreased MCS after three years in both genders, but decreased PCS after three years only in women. Thus, a greater impact of loneliness on the health of older women can be surmised and should therefore be considered in the context of their medical care.

**Supplementary Information:**

The online version of this article contains supplementary material available 10.1007/s11136-021-03055-1.

## Plain English summary

Health-Related Quality of Life (HRQOL) is influenced by physical and mental health, but also by social factors like loneliness. Older women and men differ in their social network. Still, studies examining the association of loneliness and HRQOL in older women and men are scarce. We examined the association of loneliness and HRQOL over three years in a sample of more than 2000 older adults in Germany- separated by gender. Data were recorded by trained study doctors in a large epidemiological study (ESTHER study). Data show that loneliness is associated with lower Quality of Life after three years (in terms of both, physical health and mental health) among women, but in men with lower Quality of Life in terms of mental health, only. Thus, a greater impact of loneliness on the health of older women can be surmised and should therefore be considered in the context of their medical care.

## Introduction

Health-related quality of life (HRQOL) comprises the perception and evaluation of a person concerning his/her biopsychosocial well-being. HRQOL in an aging population is not only influenced by the objective health status of a person, but also by coping resources and social support. It is well known that chronic diseases lead to a decline of HRQOL in older persons [1]. A current meta-analysis points out that multimorbidity is associated with lower HRQOL, and HRQOL decreases continually by every added disease [2]. Also, mental diseases such as depression or anxiety disorders have been shown to reduce several aspects of quality of life [3–5]. Other studies state that social relations, functional ability, and activities influence quality of life as much as the objective health status [6]. In this context, loneliness has shown to be associated with decreased HRQOL in older persons as well as with a greater mortality risk [7, 8]. Various studies show that women (of all age groups) report significantly lower scores of HRQOL as compared with men [9, 10]. However, the factors that determine this difference remain unclear. Social aspects such as marital quality, lower income, or poorer health status of women are discussed [9, 10]. Hajian-Tilaki et al*.* demonstrated that the adjustment for chronic disease conditions and sociodemographic factors partly explains the lower HRQOL in women, while gender differences still remain significant [11]. In this context, Baladon et al. [12] reported that anxiety and pain had an impact only on the HRQOL of women. In addition, respiratory diseases have been shown to be associated with decreased mental HRQOL of women, but with physical HRQOL in men (measured by PCS/MCS, physical/mental component score of the SF-12) [12]. Another recent publication emphasized that regarding quality of life, older women would benefit more from active social participation whereas men would benefit more from social networks and social support [13].

More recently in older persons, the influence of loneliness (the subjective and unpleasant feeling that social interactions are deficient [14]) and social isolation (objectively weak structural and functional social relationships [15]) on both health and HRQOL became of particular interest [7, 16]. Loneliness in older adults is frequent in Germany. Recently, a large epidemiologic study estimated the prevalence of loneliness with 10.5% in adults aged 35 to 74 years. Prevalence of loneliness was higher in older women in comparison with men, but particularly high in older persons who are living alone [17]. Additional variables that have shown to be associated with loneliness are as follows: older age, poor health, and low income; however, psychological factors such as low self-efficacy beliefs, negative life events, and a low level of personal resources are also determinants [18, 19]. A recent review describes social connectedness – as a possible opposite of loneliness—as a key element to health and well-being [20]. Our own previous research showed that women more frequently report being lonely, even if their social network does not differ from the network of older men [19].

Based on the observation that older women and men experience loneliness differently, we put forward the hypothesis that loneliness could have a gender-specific effect on HRQOL. The aim of this study was, therefore, to determine the longitudinal association between loneliness, social isolation, and HRQOL (mental component score (MCS) and physical component score (PCS)) in older people, separated by gender.

## Methods

### Study sample

The data stem from the third and fourth follow-up of the ESTHER study. The ESTHER study is a population-based cohort study of the older population in Germany. Its intention is epidemiological research on prevention, early recognition, and medical care of chronic diseases [21, 22]. The study population (*n* = 9953 at baseline) was recruited by general practitioners in the Federal State of Saarland (from July, 2000, to December, 2002) following a health check-up that is offered to people age 35 years and older in Germany. At baseline, the ESTHER study sample was shown to be representative of the general German population [22]. At the third (8-year) follow-up of the ESTHER study, between 2008 and 2010, a total of 6063 older persons took part. Of these ESTHER participants, 3124 attended a home visit (i.e., the first home visit of the ESTHER study), led by trained study doctors. The home visit included an extensive assessment of functional status as well as of the participants’ medical, psychosocial, and socioeconomic features. Between 2011 and 2013, the fourth (11-year) follow-up of the ESTHER study was conducted that included a second home visit; equivalent data on biopsychosocial health were recorded by the study doctors with the use of standardized interviews. Participants for whom complete data on health-related quality of life and loneliness were obtained at the first (t0) and second (t1) home visit (i.e., third and fourth follow-up of the ESTHER study) were then included in the current analysis.

### Measurements

#### Health-related Quality of Life (HRQOL)

Health-related quality of life was assessed by using the short-form general health survey (SF-12) [23]. The SF-12 is a patient-reported survey that records information on physical and mental quality of life according to 12 items. It is a reduced version of the SF-36 and has shown good psychometric criteria for the use in large epidemiological settings [24]. HRQOL is measured by physical (PCS) and mental (MCS) composite scores that correlate with physical and mental well-being, each score ranging from 0 to 100. Higher scores indicate a higher quality of life. The correlation between the two baseline predictors MCS and PCS was r = 0.21 in our data. This low correlation is due to the algorithms that calculate the two scores.

#### Social health

Loneliness was measured by using a three-item questionnaire based on the revised UCLA (University of California, Los Angeles) Loneliness Scale, validated for use in large epidemiological settings [25, 26]. It is composed of three items: “How often do you feel lonely?”, “How often do you feel that you lack companionship?”, and “How often do you feel left out?”. The response categories were coded 1 (hardly ever), 2 (sometimes), and 3 (often). A total score was calculated, with the higher scores representing greater loneliness. Prevalence of loneliness was estimated by a score of seven and greater: participants were classified as having a high degree of loneliness, meaning that they responded to at least one of the questions with “often” and to the other questions with “sometimes.” Social isolation (perceived social support received by family and friends) was assessed by using the Lubben Social Network Scale-6 (LSNS-6) [27] that is a six-item, self-reported scale to assess social isolation in older adults. The LSNS-6 measures the frequency and quality of contacts of a participant`s social network (including family and unrelated persons). The score ranges from 0 to 30 with a cut-off of less than 12 for defining individuals as being socially isolated [27]. We divided the sample into two subgroups in accordance with this cut-off.

#### Mental health

Severity of depression, somatization disorder, and generalized anxiety disorder were measured by using questionnaires; depression was measured with the German Patient Health Questionnaire (PHQ-8 version). The PHQ-8 consists of eight of the nine DSM-IV criteria on which the diagnosis of depressive disorders is based [28]. The ninth criterion, which asks for suicidal ideation, was left out. The PHQ-8 was shown to be a useful and valid depression measure in large population-based setting. Current depression was defined by the following algorithm: a minor or major depression was diagnosed if (1) 2–4 or ≥ 5 of the 8 items were present on “more than half the days” and one item was anhedonia or depressed mood or (2) if patients had a PHQ-8 score > 10. Somatization disorder was measured by an adapted version of the PHQ-15 with 13 questions, including questions about physical pain [29]. Questions concerning problems during menstruation or sexual intercourse were omitted. Participants with a somatic symptom score ≥ 13 were categorized as having “somatization disorder” [29], while generalized anxiety disorder was assessed by using the GAD-7 [30]. Patients with a GAD-7 > 5 were shown to fulfill the criteria for generalized anxiety disorder in older adults [31].

#### Physical health

Physical health was estimated by the prevalence of chronic diseases. It was assessed with the chronicity-variable of the somatic domain of the INTERMED for the elderly (IM-E) interview [32]—an integrative interview to identify patients with complex health care needs. The variables range from 0 to 3, with 0 reflecting good health and 3 reflecting serious illness and a high need. The chronicity-variable of the IM-E asks about the existence of somatic diseases: “Which of your physical illnesses have been ascertained over the last 5 years?”. Participants were accordingly categorized into three subgroups. Participants assessed as having no chronic disease formed subgroup 1; participants assessed as having “one chronic disease” were classified in subgroup 2; subgroup 3 were the participants with “several chronic diseases” or “multimorbidity.”

### Statistical analysis

Participants were included in the analysis if they completed the data of the SF-12 and loneliness in the third (t0: first home visit) and fourth follow-up (t1: second home visit) of the ESTHER study. First, the study sample was characterized by descriptive statistics; mean scores and confidence intervals of MCS and PCS were then calculated and compared by using t-tests, separately for women and men, stratified by loneliness. Finally, gender-specific linear regression analyses were performed to predict PCS and MCS after three years. In the final linear regression models, age, marital status, education, physical health (prevalence of chronic diseases), mental health (severity of depression symptoms, somatic symptoms or anxiety), loneliness (severity), social isolation (subgroups), and PCS and MCS at t0 were included. A *p*-value of < 0.05 in two-sided testing was considered significant. For each variable, standardized regression coefficients were calculated (“*β*”) to permit direct comparisons of the influence on PCS/MCS at t1 among predictor variables. Standardized regression coefficients are scale-free parameters that can be used to compare the magnitude of effects across studies. The standardized regression coefficient is interpreted as the estimated number of standard deviations of change in the dependent variable for one standard deviation unit change in the independent variable, controlling for other independent variables. As MCS and PCS have been shown to be the strongest predictors for MCS and PCS at a later time point [34, 35], we have run hierarchical linear regressions with first including MCS and PCS at baseline, and then entering all the other variables of interest as predictors for MCS and PCS three years later. Determination coefficients (R^2^) were calculated and reported for both models (basic model including only MCS and PCS at baseline vs. full model). We controlled results of linear regression analyses for multiple comparisons by calculating Bonferroni-corrected *p*-values and *p*-values based on the false discovery rate approach (Online Appendix 1a–d). All regression models were tested for multicollinearity using the collinearity diagnostics of SAS, proc reg. The condition indices of the four regression models were not large indicating low or acceptable correlation among variables.

Statistical analysis was performed using SAS, version 9.4.

## Results

2171 participants (1129 female, 1042 male) took part in both home visits [third (t0) and fourth follow-up (t1) of the ESTHER study], completed the SF-12 and the 3-item UCLA loneliness questionnaire, and were therefore included in the study. Table 1 shows the demographic characteristics of the study sample at the third follow-up of the ESTHER study (t0; 2008–2010).

**Table 1:** Demographic characteristics of the study population at the third follow-up of the ESTHER study (2008–2010)

Baseline variables	Women			Men			*p* (Chi^2^)
	*n*	(%)	95%CI	*n*	(%)	95%CI	
Age (years)							
55–64	326	28.9	26.5.; 31.6	253	24.3	21.7; 27.0	.043
65–74	602	53.3	50.4; 56.3	579	55.6	52.5; 58.6	
75–84	201	17.8	15.6; 20.2	210	20.2	17.8; 22.8	
Education (years)*							
< 9	11	1.0	0.5; 1.8	14	1.4	0.8; 2.3	< .001
9–10	981	88.2	86.2; 90.1	762	74.1	71.3; 76.7	
11–12	53	4.8	3.5; 6.0	141	13.7	11.7; 16.0	
> 12	67	6.0	4.7; 7.6	112	10.9	9.1; 13.0	
Marital status**							
Single	41	3.6	2.6; 4.9	37	3.6	2.5; 4.9	< .001
Married	693	61.5	58.6; 63.4	889	85.8	83.5; 87.9	
Divorced/widowed	393	34.9	31.1; 37.7	110	10.6	8.8; 12.7	
Physical health***							
No chronic disease	299	26.5	23.9; 29.2	284	27.3	24.6; 30.1	.92
1 chronic disease	245	21.7	19.3; 24.2	223	21.4	19.0; 24.0	
≥ 2 chronic diseases	585	51.8	48.9; 54.8	534	51.3	48.2; 54.4	
Mental health							
Depression	104	9.3	7.6; 11.1	48	4.6	3.4; 6.1	< .001
Somatization	88	7.8	6.3; 9.5	27	2.6	1.7; 3.8	< .001
GAD	54	4.8	3.6; 6.2	18	1.8	1.0; 2.7	< .001
Social network							
Loneliness(high degree)	215	19.0	16.8; 21.5	79	7.6	6.1; 9.4	< .001
Social isolation	133	11.8	10.0; 13.8	154	14.8	12.7; 17.1	.039

Total: 2171 participants; *CI* confidence interval; *p* Chi^2^-test comparing women and men

*Missing = 30; **missing = 8; ***missing = 1;

Older women and men showed significant differences regarding age, education, marital status, prevalence of mental diseases, loneliness, and social isolation, but showed no significant difference regarding physical health (Please see Table 1).

At t0, data on loneliness and HRQOL was obtained from 3026 participants. Of these, 2171 participants also answered questions on loneliness and HRQOL at t1 and were included in the regression analyses of the present study. *n* = 855 persons did not complete the questions at t1 (due to various reasons such as withdrawal of the study, death, or non-compliance) and were excluded from the study. Compared to study participants, persons excluded from the study showed significantly lower MCS, PCS, and LSNS-6 scores at t0. Persons excluded from the study were significantly older in comparison with study participants (71.0 years. (SD: 6.5) vs. 69.1 years. (SD: 6. 1); *p* < 0.001). No differences between groups were observed regarding age and the UCLA loneliness score at t0.

### Health-related Quality of Life—separated by gender

The MCS-score was significantly lower in older women as compared with men at t0 and t1 (*p* < 0.001/*p* < 0.001). The PCS-score was significantly lower in women as compared with men at t0 (*p* = 0.04), while there was no significant difference at t1 (*p* = 0.12). In both genders, MCS- and PCS-scores were lower after three years in comparison with t0 (Please see Table 2 for details.)

**Table 2:** HRQOL—separated by gender at the third and fourth follow-up of the ESTHER study

	Third follow-up (t0)		Fourth follow-up (t1)		
	Mean	SD	Mean	SD	Difference t1-t0
*MCS*					
Total	48.3	9.5	47.4	10.0	− .87
Women	47.0	10.2	46.0	10.6	− 1.01
Men	49.6	8.6	48.9	9.2	− .73
*PCS*					
Total	41.7	9.8	40.7	10.0	− 1.03
Women	41.3	10.0	40.4	10.1	− .93
Men	42.2	9.6	41.1	9.9	− 1.14

### Loneliness prevalence

294 participants (13.5%; 95%-CI = [12.1; 15.1]) were estimated to be lonely according to the 3-item UCLA loneliness scale at the first home visit. 36.1% (CI = [30.6; 41.7]) of persons who were lonely at the first home visit were also lonely at the second home visit. Loneliness prevalence at the second home visit was 9.0% (CI = [7.9; 10.0]). There was a higher proportion of loneliness in female participants (t0: (19.0% vs 7.6%; *p* < 0.001)) in comparison with male participants and in persons who are living alone or divorced compared to married individuals (*p* < 0.001). There was no significant difference regarding age groups (*p* = 0.18) in lonely and less lonely participants. In lonely persons, 24.1% (CI = [19.8; 28.9]) also reported being socially isolated. In lonely women, the number of persons who reported social isolation was significantly lower (19.2% (CI = [14.9; 25.0]) as compared with lonely men (36.9% (CI = [26.6; 48.1]) (*p* = 0.001).

### HRQOL in lonely and less lonely participants

**Fig. 1:**
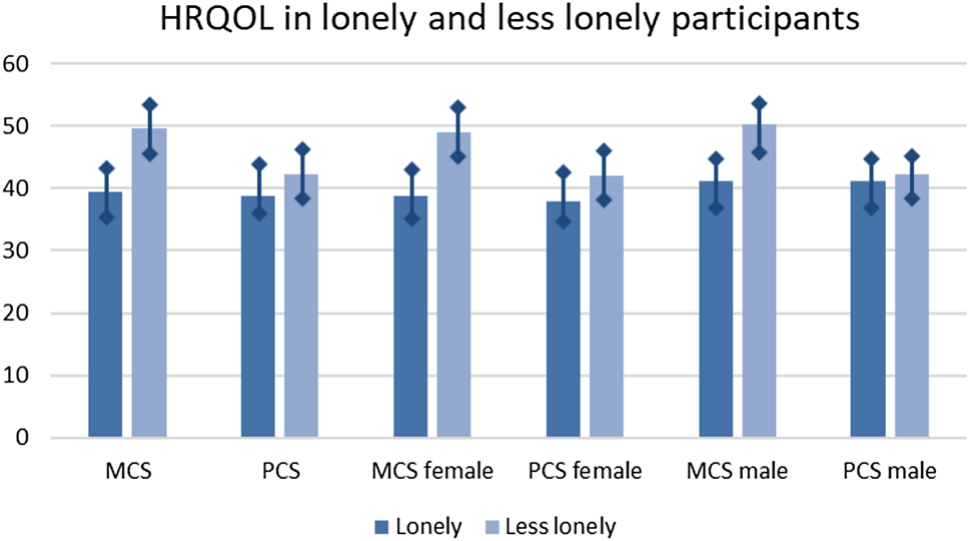
HRQOL at t0 in older women and men, separated by loneliness subgroups. Significant difference comparing lonely and less lonely participants in all categories except for PCS male

At t0, MCS was significantly lower in lonely participants in comparison with less lonely participants in both genders (*p* < 0.001). PCS was significantly lower in lonely women in comparison with less lonely women (*p* < 0.001) but not in men (*p* = 0.35). (Fig. 1 provides an overview.)

### Longitudinal predictors of HRQOL

To predict HRQOL at t1, we conducted four different linear regression analyses with PCS and MCS as dependent variables (separated by gender). Please see Table 3 for the results of multiple regression analysis of PCS over time in older women and men. Bonferroni-corrected and false discovery rate *p*-values for each variable are presented in Online Appendix 1(a–d).

**Table 3:** Multiple linear regression analysis with several independent baseline variables of biopsychosocial health predicting PCS 3 years later, separated by gender

Baseline variables	Women				Men			
	*B*^a^	SE^b^	*β*^c^	*p*-value	*B*^a^	SE^b^	*β*^c^	*p*-value
Age (years)								
55–64	**1.26**	**.49**	**.06**	**.011**	**2.25**	**.55**	**.10**	** < .001**
65–74 (ref.^d^)								
75–84	**− 2.02**	**.58**	**− .08**	**.001**	**− 1.33**	**.59**	**− .05**	**.024**
Education (years)								
0–8	− .42	2.13	< -.01	.834	− 2.06	1.95	− .02	.291
9–10 (ref.)								
≥ 11	− 1.15	1.00	− .02	.249	− .73	.66	− .03	.267
11–12	.41	.89	.01	.643	.76	.73	.02	.300
Marital status								
Single	− .34	1.15	− .01	.765	− 1.16	1.26	− .02	.356
Married (ref.)								
Divorced/widowed	− .16	.46	− .01	.731	.25	.75	.01	.743
Physical health								
No chronic disease	**1.07**	**.53**	**.05**	**.044**	**2.12**	**.55**	**.10**	** < .001**
1 chronic disease	**1.14**	**.54**	**.05**	**.037**	.48	.58	.02	.404
≥ 2 chronic diseases (ref.)								
Mental health								
Somatic symptoms_t0_	**− 3.38**	**.89**	**− .09**	** < .001**	− 1.81	1.54	− .03	.239
Depression symptoms_t0_	.91	.88	.03	.304	− 1.34	1.25	− .03	.289
GAD symptoms_t0_	.74	1.11	.02	.503	1.61	1.86	.02	.388
Social health								
Loneliness_continuously_	**− .33**	**.13**	**− .06**	**.008**	.22	.17	.03	.190
Social network_large_	− .98	.67	− .03	.146	**− 1.68**	**.65**	**− .06**	**.010**
HRQOL								
MCS_t0_	**.06**	**.03**	**.07**	**.010**	**.10**	**.03**	**.08**	**.001**
PCS_t0_	**.63**	**.02**	**.63**	**.010**	**.65**	**.03**	**.62**	** < .001**

Significant associations are printed in bold, variables with gender differences are framed

^a^Non-standardized regression coefficient

^b^Standard error

^c^Standardized regression coefficient

^d^Referent

**Table 4:** Multiple linear regression analysis with several independent baseline variables of biopsychosocial health predicting MCS three years later, separated by gender

Baseline variables	Women				Men			
	*B*^a^	SE^b^	β^c^	*p*-value	B^a^	SE^b^	β^c^	*p*-value
Age (years)								
55–64	0.29	.56	.01	.603	.27	.56	.01	.634
65–74 (ref.^d^)								
75–84	− 1.16	.66	− .04	.080	− .84	.60	− .04	.162
Education (years)								
0–8	− 4.24	2.43	− .04	.081	1.15	1.99	.02	.562
9–10 (ref.)								
≥ 11	− .28	1.14	− .01	.808	− .031	.67	< -.01	.964
11–12	.76	1.01	.02	.451	1.06	.75	.04	.159
Marital status								
Single	**2.83**	**1.31**	**.05**	**.031**	1.07	1.28	.02	.404
Married (ref.)								
Divorced/widowed	.89	.53	.04	.093	1.02	.77	.03	.183
Physical health								
No chronic disease	− .01	.61	< -.01	.989	.61	.56	.03	.275
1 chronic disease	.46	.62	.02	.455	.11	.59	.01	.855
≥ 2 chronic diseases (ref.)								
Mental health								
Somatization symptoms _t0_	− .91	1.02	− .02	.371	− 2.83	1.57	− .05	.071
Depression symptoms_t0_	**− 2.03**	**1.00**	**− .06**	**.043**	− .68	1.28	− .02	.593
GAD symptoms_t0_	− .93	1.27	− .02	.464	− 3.30	1.90	− .05	.083
Social health								
Loneliness_continuously_	**− .29**	**.14**	**− .05**	**.043**	**− .45**	**.17**	**− .07**	**.008**
Social network_large_	− .79	.77	− .02	.306	− .55	.67	− .02	.408
HRQOL								
MCS_t0_	**.55**	**.03**	**.54**	** < .001**	**.51**	**.03**	**.48**	** < .001**
PCS_t0_	**.17**	**.03**	**.16**	** < .001**	**.18**	**.03**	**.18**	** < .001**

Significant associations are printed in bold, variables with gender differences are framed)

^a^Non-standardized regression coefficient

^b^Standard error

^c^Standardized regression coefficient

^d^Referent

In women, younger age, good physical health (having none or one chronic disease), and MCS and PCS at t0 were positively associated with PCS after 3 years (t1); PCS at t1 was negatively associated with loneliness at t0, older age, and somatic symptoms. The strongest predictor of PCS after three years in women were PCS at t0 (*p* < 0.001) and somatic symptoms (*p* = 0.002). In men, younger age, good physical health (no chronic disease at t0), and MCS and PCS at t0 were positively associated with PCS after 3 years; PCS at t1 was negatively associated with older age and a large social network. The strongest predictors of PCS after 3 years were PCS at t0 (*p* < 0.001) and younger age (*p* < 0.001).

In women, the determination coefficient R^2^ for the model including only MSC and PCS at baseline was *R*^2^ = 50, R^2^ for the complete regression model was 0.53. In men, R^2^ for the model including only MCS and PCS at baseline was 0.46, and R^2^ for the complete regression model was 0.49.

In women, being single, and MCS and PCS at t0 were positively associated with MCS after three years; depression symptoms and loneliness at t0 were negatively associated with MCS at t1. The strongest predictors for MCS at t1 in women were MCS and PCS at t0 (*p* < 0.001/*p* < 0.001). In older men, MCS and PCS at t0 were positively associated with MCS after three years. It was negatively associated with loneliness, but not depression symptoms. The strongest predictors for MCS at t1 in men were MCS and PCS at t0 (*p* < 0.001/*p* < 0.001). In women, the determination coefficient (R^2^) for the basic model (MCS and PCS at baseline as predictors) was 0.43, and R^2^ for the full regression model was 0.44. In men, R^2^ for the basic model was 0.5, and R^2^ for the full regression model was 0.37 (Table 4).

Analyzing a possible reverse causation with loneliness at t1 as predictor variable and MCS/PCS at t0 as independent variables in women, results of the multiple linear regression analysis showed that increased loneliness at t1 was predicted by older age, being divorced/widowed at t0, loneliness at t0, and MCS and PCS at t0. In older men, however, increased loneliness at t1 was predicted by being single/divorced/widowed at t0, somatization symptoms at t0, a small social network at t0, and MCS, but not PCS at t0.

## Discussion

Our data show that HRQOL in older women and men is predicted by different biopsychosocial factors. While loneliness predicts decreased MCS in both genders, it predicts decreased PCS after three years in women only. These observations are an interesting addition for the field of HRQOL—research in older persons and remind us of the need to keep gender aspects in mind when treating older persons.

Loneliness has been shown to be associated with decreased HRQOL in several studies [7]. Tan et al. observed that both emotional and social loneliness were associated with lower mental and physical HRQOL but found a particularly large difference in lonely and non-lonely participants regarding mental HRQOL [33]. Vespa et al. furthermore demonstrated that loneliness, also in presence of interpersonal relations, predicted an inferior quality of life in persons suffering from multimorbidity [34]. However, none of these studies focused on gender-specific differences.

Thus, what could be the cause for the gender-specific association between loneliness and HRQOL?

First of all, loneliness in older women is more frequent and often more severe [17]. Older women were shown to feel lonely more frequently in comparison with men, even if there is no difference in the quantity or quality of their social network [19]. Thus, it can be hypothesized that women are more sensitive regarding feelings of loneliness or social dissonance. In addition, David-Barrett et al. described that women rely more on close one-to-one relationships in comparison with men [35]. In this context, Li et al. [36] revealed that several types of social activities showed weaker associations with HRQOL among women as compared with men. Our own previous work showed, furthermore, that older men reported free-time activities significantly more frequently as something that gives them strength as compared with women [37]. On the other hand, a recent study in Canada found that women and men do not differ significantly in terms of desired and actual social participation [38].

Furthermore, from our data, it is interesting that a stronger social network was a predictor for decreased PCS in older men. This result is counter-intuitive and not in line with previous research [39]. While, at this point, we have no explanation for this association, it also indicates that not only quantity but quality of socials contacts over time are crucial to HRQOL. Interestingly, being single was a predictor for increased MCS in older women. Here, it can be hypothesized that marital contacts could indeed also bestow distress, particularly if potential caregiving duties are involved—keeping in mind that women are disproportionally, and more often, in charge of caregiving in families [40].

However, all of these considerations only aim to explain why the influence of loneliness on HRQOL in general might be greater in women. One primary observation from our data is that in older women loneliness is associated with decreased physical HRQOL. Murtagh et al. found that older women report functional limitations and disabilities more often, but also that these gender differences were largely explained by poorer health conditions in women [41]. This is partly contradictive to our data which show a similar degree of physical health for older women and men (see Table 1). Another possibility is that loneliness in women limits access to health care, resulting in decreased PCS over time. In this context, it has been shown that women—who more frequently live alone in older age—are more likely to experience transportation problems in comparison with men [42–44]. However, in our data, for women, the size of the social network is not associated with PCS. This indicates that for older women, a higher quality but not the quantity of social support or networks are associated with an increased physical HRQOL three years later. This is also reflected in the finding that only 24.1% of people categorized as lonely reported social isolation.

At this point, we must emphasize that our explanations for the longitudinal association of loneliness and PCS in older women and men remain somewhat speculative. Geographic and cultural criteria must be taken into consideration. Furthermore, we have to point out that reverse causation might be possible. Lower HRQOL could lead to a more negative assessment of social relationships which could result in the reporting of greater loneliness. Our data show that in women, loneliness at t1 was predicted by MCS and PCS at t0, while in men, loneliness at t1 was predicted by MCS, but not PCS at t0. Following the hypothesis that HRQOL could shape the assessment of social relationships, gender differences in how older women and men assess well-being—and the possible future consequences regarding social interaction and contact–become evident and may open an interesting field for further research.

Another finding is that MCS and PCS at baseline were the strongest predictors for MCS—equally in both genders. This corresponds with previous research, for instance, by Brett et al. showing that HRQOL, at a specific point in time, is a good and stable predictor for HRQOL over a period of time [44, 45]. Furthermore, our observation of the association of multimorbidity and older age with lower HRQOL is supported by other studies [2]. This also explains why MCS and PCS were lower at t1 in comparison with t0 which is also described in other samples with older person, for example, by Webb et al. [46].

In addition, we found that MCS and PCS (at t0) were significantly lower in older women as compared with men. This corresponds with other studies conducted in an older population [47]. Interestingly, a cross-sectional survey in 6 European countries described that German respondents rated their mental health above the mean score in comparison with participants from, for example Italy or France, but rated their physical health below the mean, independent of age, education, living situation, or employment status [48]. From our data, loneliness prevalence can be estimated with 13.5%. It was more frequent in women and in persons that are living alone, but was not associated with age groups; lonely women reported social isolation more frequently than lonely men. The loneliness prevalence corresponds with data from Domenech-Abella who describe a preponderance of loneliness in women and in persons who are living alone, but also an association of loneliness and younger age [49]. Similar results are reported by Beutel et al. [17].

Our study has several limitations. Firstly, it is necessary to point out that we recruited a relatively healthy sample of older adults and did not include nursing home or retirement home residents; this must be kept in mind when interpreting our data. Secondly, mental diseases were examined by using questionnaires; regardless, the PHQ-9, PHQ-15, and the GAD-7 were shown to have good psychometric properties and were well applicable in population-based studies [50]. Thirdly, the lack of intervening variables in our data collection do not allow for investigating the potential mechanisms underlying the observed relationships. Thus, our results remain speculative to a certain degree. The particular strength of this study is the sample size of the study population (*n* = 2171). In addition, data were obtained during a comprehensive home visit over a period of several hours, conducted by trained study doctors. The study doctors recorded and assessed chronic diseases and multimorbidity by well-validated measures. This provided us with data of high quality in a large setting of older adults.

In conclusion, the improvement of well-being in older persons does not equal to optimize physical health by treating disease by disease, but aiming to promote HRQOL by looking at all dimensions of biopsychosocial health including gender. The most important finding of our study is that biopsychosocial predictors for HRQOL differ in older women and men. Purportedly, loneliness has a greater impact on the HRQOL of older women because it predicts lower PCS in older women, but not in older men. This underlines the importance of social connectedness, with and without help of institutions for social care, for the promotion of health and well-being. Furthermore, the longitudinal association of loneliness and lower MCS in both genders—as described previous research—is supported by our data. This underlines the need to assess social factors such as loneliness in older persons and to target loneliness as a health care problem, not only—but especially—in older women.

## Supplementary Information

Below is the link to the electronic supplementary material.

Electronic supplementary material 1 (DOCX 31 kb)

## Abbreviations

HRQOL: Health-related quality of life
ESTHER: Epidemiologische Studie zu Chancen der Verhütung, Früherkennung und optimierten THerapie chronischer ERkrankungen in der älteren Bevölkerung
SF-12: Short-form health survey
MCS: Mental composite score (of the SF-12)
PCS: Physical composite score (of the SF-12)
PHQ: Patient health questionnaire
GAD: Generalized anxiety disorder
IM-E: INTERMED for the elderly
