# Supplementary material for: Loneliness as a gender-specific predictor of physical and mental health-related quality of life in older adults
Source: Qual Life Res. 2021 Dec 2;31(7):2023–33. doi: 10.1007/s11136-021-03055-1 (PMC9188519; doi:10.1007/s11136-021-03055-1)
Supplement: Supplementary file 1 — Electronic supplementary material 1 (DOCX 31 kb) [file 11136_2021_3055_MOESM1_ESM.docx]

**Appendix 1a: Multiple regression analysis predicting MCS 3 years later in women, controlled for multiple comparison**

| **Baseline variables** | **raw**  **p- value** | **Bonferroni**  **p-value** | **false discovery rate**  **p value** |
| --- | --- | --- | --- |
| Age (years) |  |  |  |
| 55-64 | .603 | 1.000 | .689 |
| 65-74 (ref. ^4^) |  |  |  |
| 75-84 | .080 | 1.000 | .185 |
| Education (years) |  |  |  |
| 0-8 | .081 | 1.000 | .185 |
| 9- 10 (ref.) |  |  |  |
| ≥11 | .808 | 1.000 | .862 |
| 11-12 | .451 | 1.000 | .571 |
| Marital status |  |  |  |
| Single | **.031** | .494 | .139 |
| Married (ref.) |  |  |  |
| Divorced / widowed | .093 | 1.000 | .186 |
| Physical health |  |  |  |
| No chronic disease | .989 | 1.000 | .989 |
| 1 chronic disease | .455 | 1.000 | .571 |
| ≥2 chronic diseases (ref.) |  |  |  |
| Mental health |  |  |  |
| Somatic symptoms _t0_ | .371 | 1.000 | .571 |
| Depression symptoms _t0_ | **.043** | .693 | .139 |
| GAD symptoms _t0_ | .464 | 1.000 | .571 |
| Social health |  |  |  |
| Loneliness _continuously_ | **.043** | .685 | .139 |
| Social network _large_ | .306 | 1.000 | .544 |
| HRQOL |  |  |  |
| MCS _t0_ | **<.001** | **.0016** | **.0008** |
| PCS _t0_ | **<.001** | **.0016** | **.0008** |

**Appendix 1b: Multiple regression analysis predicting MCS 3 years later in men, controlled for multiple comparison**

| **Baseline variables** | **raw**  **p- value** | **Bonferroni**  **p-value** | **false discovery rate**  **p value** |
| --- | --- | --- | --- |
| Age (years) |  |  |  |
| 55-64 | .634 | 1.000 | .725 |
| 65-74 (ref. ^4^) |  |  |  |
| 75-84 | .162 | 1.000 | .367 |
| Education (years) |  |  |  |
| 0-8 | .562 | 1.000 | .725 |
| 9- 10 (ref.) |  |  |  |
| ≥11 | .964 | 1.000 | .964 |
| 11-12 | .159 | 1.000 | .367 |
| Marital status |  |  |  |
| Single | .404 | 1.000 | .594 |
| Married (ref.) |  |  |  |
| Divorced / widowed | .183 | 1.000 | .367 |
| Physical health |  |  |  |
| No chronic disease | .275 | 1.000 | .488 |
| 1 chronic disease | .855 | 1.000 | .912 |
| ≥2 chronic diseases (ref.) |  |  |  |
| Mental health |  |  |  |
| Somatic symptoms _t0_ | .071 | 1.000 | .266 |
| Depression symptoms _t0_ | .593 | 1.000 | .725 |
| GAD symptoms _t0_ | .083 | 1.000 | .266 |
| Social health |  |  |  |
| Loneliness _continuously_ | **.008** | .134 | **.045** |
| Social network _large_ | .408 | 1.000 | .594 |
| HRQOL |  |  |  |
| MCS _t0_ | **<.001** | **.0016** | **.0008** |
| PCS _t0_ | **<.001** | **.0016** | **.0008** |

**Appendix 1c: Multiple regression analysis predicting PCS 3 years later, in women controlled for multiple comparison**

| **Baseline variables** | **raw**  **p- value** | **Bonferroni**  **p-value** | **false discovery rate**  **p value** |
| --- | --- | --- | --- |
| Age (years) |  |  |  |
| 55-64 | **.011** | .173 | **.029** |
| 65-74 (ref. ^4^) |  |  |  |
| 75-84 | **.001** | .008 | **.004** |
| Education (years) |  |  |  |
| 0-8 | .834 | 1.000 | .843 |
| 9- 10 (ref.) |  |  |  |
| ≥11 | .249 | 1.000 | .398 |
| 11-12 | .643 | 1.000 | .791 |
| Marital status |  |  |  |
| Single | .765 | 1.000 | .816 |
| Married (ref.) |  |  |  |
| Divorced / widowed | .731 | 1.000 | .816 |
| Physical health |  |  |  |
| No chronic disease | **.044** | .702 | .088 |
| 1 chronic disease | **.037** | .589 | .084 |
| ≥2 chronic diseases (ref.) |  |  |  |
| Mental health |  |  |  |
| Somatic symptoms _t0_ | **<.001** | .003 | .003 |
| Depression symptoms _t0_ | .304 | 1.000 | .442 |
| GAD symptoms _t0_ | .503 | 1.000 | .671 |
| Social health |  |  |  |
| Loneliness _continuously_ | **.008** | .126 | **.029** |
| Social network _large_ | .146 | 1.000 | .260 |
| HRQOL |  |  |  |
| MCS _t0_ | **.010** | .165 | **.029** |
| PCS _t0_ | **.010** | .165 | **.029** |

**Appendix 1d: Multiple regression analysis predicting PCS 3 years later, in men controlled for multiple comparison**

| **Baseline variables** | **raw**  **p- value** | **Bonferroni**  **p-value** | **false discovery rate**  **p value** |
| --- | --- | --- | --- |
| Age (years) |  |  |  |
| 55-64 | **<.001** | **.002** | **.001** |
| 65-74 (ref. ^4^) |  |  |  |
| 75-84 | **.024** | .378 | **.063** |
| Education (years) |  |  |  |
| 0-8 | .291 | 1.000 | .401 |
| 9- 10 (ref.) |  |  |  |
| ≥11 | .267 | 1.000 | .401 |
| 11-12 | .300 | 1.000 | .401 |
| Marital status |  |  |  |
| Single | .356 | 1.000 | .431 |
| Married (ref.) |  |  |  |
| Divorced / widowed | .743 | 1.000 | .743 |
| Physical health |  |  |  |
| No chronic disease | **<.001** | **.002** | **.001** |
| 1 chronic disease | .404 | 1.000 | .431 |
| ≥2 chronic diseases (ref.) |  |  |  |
| Mental health |  |  |  |
| Somatic symptoms _t0_ | .239 | 1.000 | .401 |
| Depression symptoms _t0_ | .289 | 1.000 | .401 |
| GAD symptoms _t0_ | .388 | 1.000 | .431 |
| Social health |  |  |  |
| Loneliness _continuously_ | .190 | 1.000 | .401 |
| Social network _large_ | **.010** | .163 | **.033** |
| HRQOL |  |  |  |
| MCS _t0_ | **.001** | **.016** | **.004** |
| PCS _t0_ | **<.001** | **.002** | **.001** |
